# New Appliances and Things Medical

**Published:** 1896-02-15

**Authors:** 


### NEW APPLIANCES AND THINGS MEDICAL.

[We shall be glad to receive, at our Office, 428, Strand, London, W.C., from the manufacturers, specimens of all new preparations and appliances, which may be brought out from time to time.]

### KUTNOW'S IMPROVED EFFERVESCENT CARLSBAD POWDER.

(KUTNOW AND Co., 66, HOLBORN VIADUCT.)

A sample of this powder has been forwarded to us for analysis and trial. Although containing the same mineral constituents which give to the natural Carlsbad waters their valuable properties, the disagreeable taste peculiar to the latter is skilfully disguised, while a slight effervescence imparts a pleasant freshness to the solution. It is undoubtedly a valuable preparation for those of gouty or rheumatic diathesis, while for the plethoric and sufferers from habitual constipation it is a pleasant and effective stand-by. While being perfectly satisfied with its therapeutic action in those for whom we have prescribed Kutnow's powder, the patients have one and all admitted that it is by no means disagreeable to the palate.

